# Analysis of Annual Variation in Stable Isotopic Fingerprints of Native Chinese Mitten Crab (*Eriocheir sinensis*) from Yangcheng Lake

**DOI:** 10.3390/ani16010028

**Published:** 2025-12-22

**Authors:** Junren Xue, Tao Jiang, Xiubao Chen, Jian Yang, Wang Zhang

**Affiliations:** 1 Freshwater Fisheries Research Center, Chinese Academy of Fishery Sciences, Wuxi 214081, China; xuejunren@ffrc.cn (J.X.);; 2 Wuxi Fisheries College, Nanjing Agricultural University, Wuxi 214081, China

**Keywords:** Chinese mitten crab (*Eriocheir sinensis*), Yangcheng lake, stable isotopic fingerprints, year-round culture period, multi-statistical analysis

## Abstract

The Chinese mitten crab (*Eriocheir sinensis*) from Yangcheng Lake is a globally renowned geographical indication product. To characterize changes in stable isotopic signatures in this species in Yangcheng Lake during the year-round culture period, this study investigated the dynamic changes in stable isotopic fingerprints (*δ*^13^C, *δ*^15^N, *δ*^2^H and *δ*^18^O) of the third pereiopod across an annual aquaculture cycle. Isotopic ratios were analyzed via one-way ANOVA, principal component analysis (PCA), linear discriminant analysis (LDA) and Pearson correlation analysis. Results demonstrated that *δ*^13^C stabilized after six months of cultivation, showing no significant temporal variation (*p* > 0.05). Multivariate statistical analysis further revealed that the samples cultured in the initial period could be clearly differentiated in both PCA and LDA plots. However, after six months, the monthly sample points became highly overlapping and indistinguishable, indicating that the crabs had developed stable and consistent isotopic “fingerprints” by that time. Pearson correlation analysis indicated significant correlations among all other isotope pairs, with the exception of *δ*^15^N and *δ*^2^H. This study confirms that isotopic signatures require prolonged cultivation (≥6 months) to reflect authentic geographical traits. In addition, our findings provide a basis for verifying the origin of Chinese mitten crab and other aquatic taxa in Yangcheng Lake.

## Simple Summary

This study investigated how the stable isotopic fingerprints of Yangcheng Lake mitten crab changes over a full year of farming. By analyzing *δ*^13^C, *δ*^15^N, *δ*^2^H and *δ*^18^O in the third pereiopod, researchers tracked these changes monthly. The key finding is that stable isotopic fingerprints of native Chinese mitten crabs need time to stabilize. While significant shifts occur in the first few months, the isotopes—particularly *δ*^13^C—become stable after six months of cultivation. After this point, the crabs’ stable isotopic fingerprints are consistent. This means that only crabs raised for at least six months in the Yangcheng Lake environment develop a true fingerprint of their origin. These findings are crucial for protecting the authentic Yangcheng Lake brand, as they provide a scientific basis to combat fraudulent products, thereby supporting the certification of premium aquatic goods.

## 1. Introduction

The Chinese mitten crab (*Eriocheir sinensis*), commonly known as the river crab or hairy crab, belongs to the family Varunidae and genus *Eriocheir*. This species is native to the eastern Pacific coastal regions of China to the Korean Peninsula, with its natural distribution spanning southeastern and northern Asia, as well as the western Korean Peninsula [1]. Notably, since the 20th century, it has progressively invaded European river systems, Mediterranean coasts, the Middle East, and the United States [2], demonstrating remarkable environmental adaptability. As a vital freshwater economic species in China, the Chinese mitten crab exhibits a broad distribution pattern: the main populations are concentrated in the Yangtze River basin, while also thriving in other major river systems such as the Ou River, Min River, Pearl River, and Liao River [3]. Its nutritional value is prominent, rich in mineral elements, crude protein, and vitamins, making it a cornerstone of China’s aquaculture industry. Leveraging its economic significance, renowned brands and export enterprise clusters, represented by the Yangcheng Lake hairy crab, have emerged [4]. According to the *2025 China Fishery Statistical Yearbook*, the total national production of Chinese mitten crabs reached 894,395 tons in 2024, with Jiangsu Province—the primary production area in the Yangtze River basin—contributing significantly, yielding 400,089 tons annually, accounting for nearly 50% of the national total [5].

The Chinese mitten crab from Yangcheng Lake in Suzhou is one of China’s most renowned geographical indication products, akin to globally celebrated delicacies such as Boston lobster. As a national geographical indication product, Yangcheng Lake Hairy Crab enjoys immense popularity among consumers. However, widespread counterfeit practices, such as ‘bathed crabs’—where non-local crabs are briefly reared in Yangcheng Lake or rinsed in its waters to fraudulently market them as authentic—have severely undermined market credibility. Noteworthily, although the actual annual production of Yangcheng Lake crabs is approximately 2000 tons and its annual sales is just about USD 42 million, the estimated annual value of market sales of Yangcheng Lake crabs even reach as much as USD 420 million, indicating that the illegal sale of counterfeit Yangcheng Lake crabs by unscrupulous vendors has severely damaged the reputation of the genuine product. Although anti-counterfeiting coding systems are widely deployed for origin protection, practical challenges persist. These include incomplete label information, physical tag loss during farming or transportation, and rampant production of counterfeit labels by unscrupulous sellers seeking illicit profits. Similar issues plague global agricultural product origin authentication, such as mislabeling of geographical indications [6,7,8,9], ambiguous origin identification [10,11], and fraudulent labeling practices [12,13], all of which jeopardize the legitimate interests of consumers, producers, and industries worldwide.

In studies on the geographical origin protection of food products, multiple analytical approaches have been developed, including geometric morphometric analysis [14,15] and elemental “fingerprint” analysis [16,17,18], both of which demonstrate robust discriminatory efficacy for differentiating food species or products from distinct origins. Among these, isotopic tracing has emerged as a widely adopted and highly accurate method in recent years. Stable isotope ratio mass spectrometry (IRMS) stands out due to its high precision, minimal sample requirements, non-radioactive nature, and pollution-free operation. This technique has seen increasing application in food product authentication [19,20,21,22]. Commonly utilized isotopic ratios for origin verification include *δ*^13^C, *δ*^15^N, *δ*^2^H and *δ*^18^O [23]. This study applied the above four stable isotopes for geographic origin tracing.

Current research on the geographical origin tracing of Chinese mitten crab from Yangcheng Lake has predominantly focused on morphological characteristics and mineral element analysis [24,25,26,27,28]. However, most aquatic product traceability studies remain limited to origin discrimination and lack systematic investigations into the formation mechanisms of geographical signatures. To address this gap, this study aims to explore the temporal dynamics of stable isotope ratios (*δ*^13^C, *δ*^15^N, *δ*^2^H and *δ*^18^O) in Yangcheng Lake crabs, specifically investigating the stabilization process of isotopic fingerprints during prolonged aquaculture. These findings will provide a theoretical foundation for safeguarding the authenticity of Yangcheng Lake crabs against fraudulent practices and serve as a methodological reference for origin authentication of other premium aquatic products globally.

## 2. Materials and Methods

### 2.1. Sample Collection

**Figure 1:**
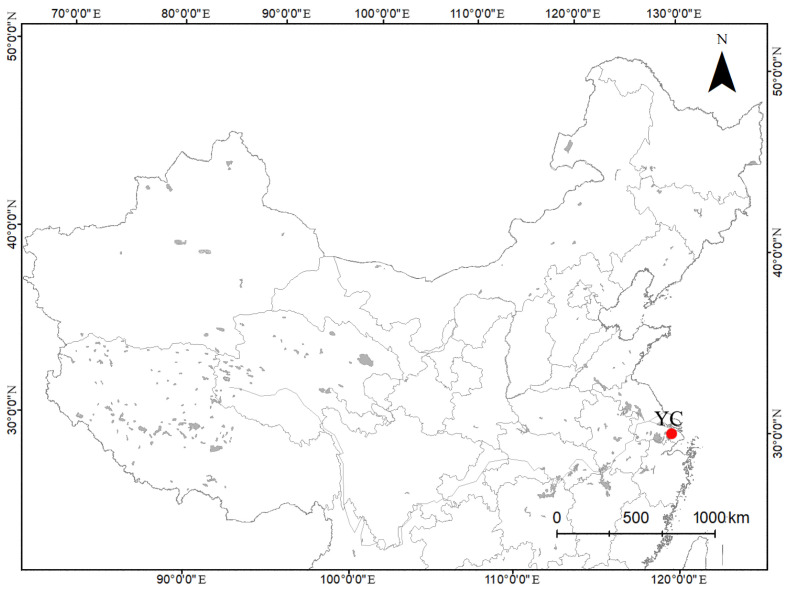
Sketch map for sampling locations of *Eriocheir sinensis*.

From March 2018 to February 2019, Chinese mitten crab samples were collected monthly from the same net-enclosed aquaculture area in Yangcheng Lake (Figure 1) (31°26.8111′ N, 120°49.4606′ E). In the study by Luo et al. [29], 20 Chinese mitten crabs were used for geographical origin traceability, achieving an accuracy rate of over 90%. Therefore, this study continues to select a sample size of 20 individuals for research. Between March 2018 and January 2019, 20 crabs (10 females and 10 males) of similar size specifications were selected each month for morphometric measurements and stable isotope ratio analysis (*δ*^13^C, *δ*^15^N, *δ*^2^H and *δ*^18^O). In February 2019, 18 crabs (8 females and 10 males) were sampled for the same analyses. All crabs originated from juvenile stocks reared at the Jiangsu Nantong Rudong Crab Seed Farm, with parent crabs from the Yangtze River population of Chinese mitten crab. In addition to consuming exogenous food sources such as fish, shellfish, and commercial pellet feed, crabs also feed on natural organisms present in water bodies. Morphometric data for all crab samples are detailed in Table 1.

### 2.2. Sample Pretreatment

Because the third pereiopod allows for non-lethal sampling while containing both exoskeletal and edible tissues, and to ensure continuity in the geographical origin tracing research of Chinese mitten crabs, all samples were uniformly processed according to the method described by Luo et al. [29], selecting the left third pereiopod for stable isotope ratio analysis. Live crabs were chilled to −20 °C for 30 min in a cabinet freezer (DW-25W388; Haier, Qingdao, China) to reduce activity. The animal study protocol was approved by the Ethics Committee of the Freshwater Fisheries Research Center, Chinese Academy of Fisheries Sciences (protocol code 2011AA1004020012, 16 January 2011). Specifically, the left third pereiopod of each specimen was rinsed six times with Milli-Q water (18.2 MΩ·cm at 25 °C; Millipore Corporation, USA) to remove surface contaminants. Subsequently, the cleaned samples were dried in an oven at 80 °C for 24 h until reaching constant weight. The dried pereiopods were then pulverized into a fine powder using an agate mortar and stored in a desiccator for subsequent isotopic analysis.

### 2.3. Stable Carbon and Nitrogen Isotope Ratio Analysis

0.2 mg of sample was weighed into a tin capsule and introduced into the isotope analyzer via an autosampler. The sample underwent combustion and reduction processes to generate purified CO_2_ and N_2_ gases, which were then analyzed using a stable isotope ratio mass spectrometer (IRMS) (Delta V Advantage, Thermo Fisher Scientific Inc., Waltham, MA, USA). The instrumental conditions were set as follows: combustion furnace temperature at 980 °C, reduction furnace temperature at 50 °C, and helium carrier gas flow rate at 100 mL/min. To ensure analytical accuracy, a laboratory reference material IAEA-600 (*δ*^13^C VPDB = −27.771 ± 0.043‰, *δ*^15^N air = 1.0 ± 0.2‰) was inserted every 10 samples for calibration. The continuous measurement precision for *δ*^13^C and *δ*^15^N was maintained at <0.06‰. If deviations in IAEA-600 values exceeded certified ranges, the system underwent gas recalibration, and affected samples were reanalyzed to correct for instrumental drift.

### 2.4. Stable Hydrogen and Oxygen Isotope Ratio Analysis

0.2 mg of sample was weighed into a silver capsule and introduced into the isotope analyzer via an autosampler. The sample underwent high-temperature pyrolysis to generate CO and H_2_ gases, which were then analyzed using a stable isotope ratio mass spectrometer (IRMS) (Delta V Advantage, Thermo Fisher Scientific Inc., Waltham, MA, USA). The instrumental parameters were set as follows: pyrolysis furnace temperature at 1380 °C, reduction furnace temperature at 75 °C, and helium carrier gas flow rate at 100 mL/min. Calibration was performed using international reference materials USGS-42 and USGS-55 via a two-point calibration method. Blank measurements from silver capsules were conducted and subtracted from experimental results to correct for background contributions. Following IUPAC guidelines [30], stable isotope ratios were calculated as: *δE* = [(*Rsample/Rstandard*)^−1^]. Where R represents the abundance ratio of heavy to light isotopes: ^13^C/^12^C, ^15^N/^14^N, ^2^H/^1^H, and ^18^O/^16^O. The *δ*^13^C values are referenced against the Vienna Pee Dee Belemnite (V-PDB) standard, *δ*^15^N values against atmospheric N_2_, and *δ*^2^H and *δ*^18^O values against the Vienna Standard Mean Ocean Water (V-SMOW).

### 2.5. Data Statistics and Analysis

Boxplots were generated using Origin 2021 (OriginLab Corporation, Northampton, MA, USA), while Linear Discriminant Analysis (LDA) and Principal Component Analysis (PCA) were also performed within the same software. One-way Analysis of Variance (ANOVA) followed by post hoc Duncan’s multiple range test was conducted in SPSS 24.0 (IBM Corporation, Armonk, NY, USA) to evaluate significant differences (*p* < 0.05) in isotopic ratios across sampling months. ANOVA was also used to examine the differences between male and female crabs. All datasets underwent normality testing. For datasets exhibiting heterogeneity of variance, Tamhane’s T2 test was applied as a non-parametric alternative [31]. Pearson correlation analyses were executed in R4.0.5.

## 3. Results

### 3.1. Analysis of Stable Isotopic Composition Differences

**Figure 2:**
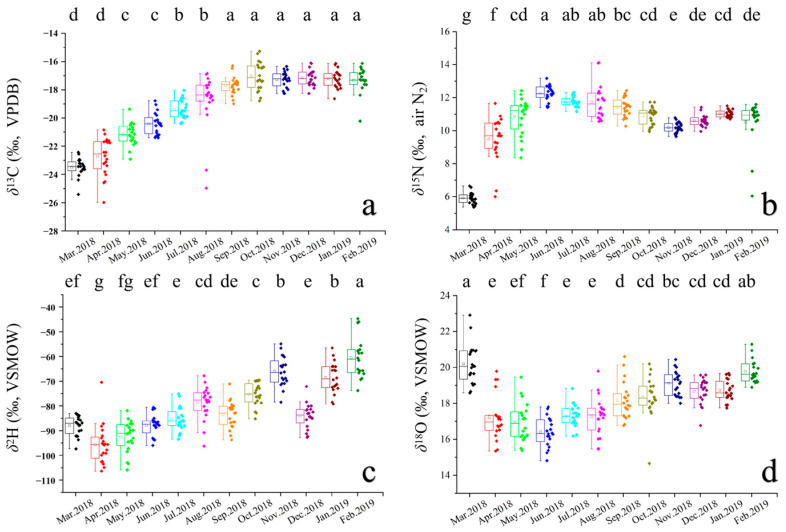
Boxplots of (**a**) *δ*^13^C, (**b**) *δ*^15^N, (**c**) *δ*^2^H, and (**d**) *δ*^18^O of crab samples. Superscript letters are significantly (*p* < 0.05) different with respect to the row for different groups.

The results of ANOVA indicated no significant difference in stable isotope values between male and female crabs in this study (*p* < 0.05); therefore, subsequent analyses were conducted without distinguishing between sexes. The stable isotope ratios (C, N, H, O) of year-round samples from Yangcheng Lake-originated Chinese mitten crabs are shown in Figure 2 and Table 2. Results of one-way ANOVA revealed that during the annual aquaculture cycle, *δ*^13^C exhibited no significant differences after six months (*p* > 0.05), while *δ*^15^N, *δ*^2^H, and *δ*^18^O showed no stable trends. Specifically, *δ*^15^N decreased significantly in November, *δ*^2^H reached its peak value in February, and *δ*^18^O displayed a pronounced trend of initial decline followed by an increase.

### 3.2. Principal Component Analysis

**Figure 3:**
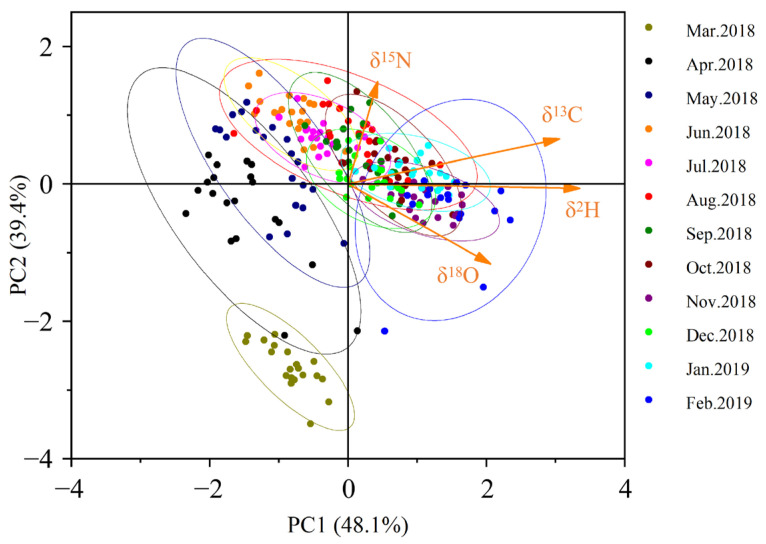
Scatter diagram of principal component analysis based on the third pereiopod stable isotopes. (95% confidence interval for ellipse).

The results of principal component analysis (PCA) on annual C, N, H, and O isotope ratios (Figure 3) revealed that the cumulative contribution rate of the first two principal components reached 87.5%, effectively explaining the variance information from the original four variables. The first principal component primarily captured the variation patterns of *δ*^13^C, *δ*^2^H, and *δ*^18^O, while the second principal component mainly reflected the variation characteristics of *δ*^15^N. During the initial aquaculture phase, cultured populations could be distinctly differentiated in the PCA scatter plot. However, a gradual stabilization process was observed, and after six months of farming, the Yangcheng Lake-originated Chinese mitten crabs could no longer be accurately distinguished in the PCA plot. This indicates that the crabs had developed stable isotopic ‘fingerprint’ characteristics.

### 3.3. Linear Discriminant Analysis

**Figure 4:**
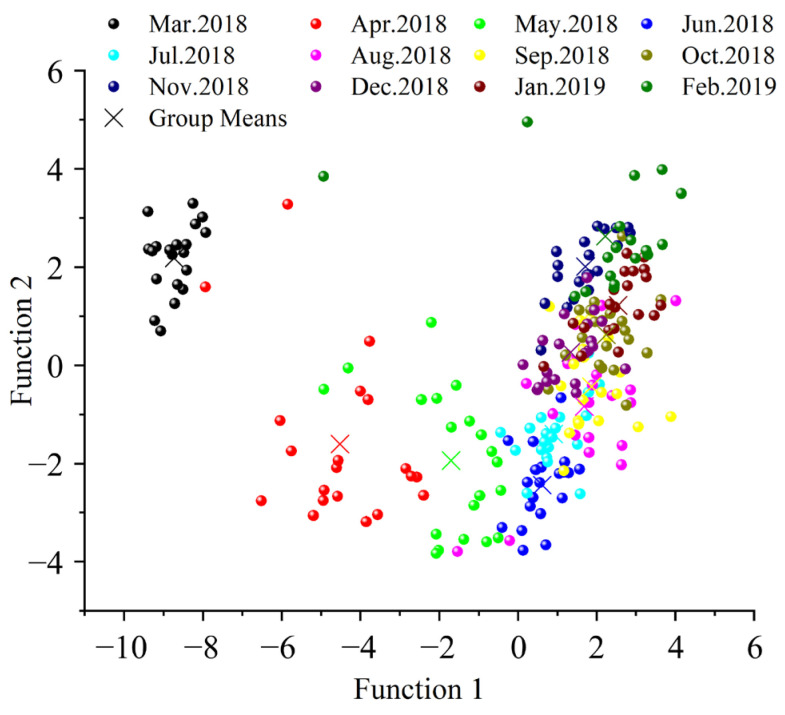
Scatter diagram of linear discriminant analysis based on the third pereiopod stable isotopes.

The results of linear discriminant analysis (LDA) on annual C, N, H, and O isotope ratios are presented in Figure 4 and Table 3. The scatter plot reveals substantial isotopic signature differences during the initial six months of aquaculture, with a stabilization trend observed over time. After July, the monthly samples in the scatter plot became highly overlapping and indistinguishable, which was further validated by the discriminant accuracy rates across months. This finding indicates that Yangcheng Lake Chinese mitten crabs began developing stable geographical isotopic ‘fingerprint’ characteristics after six months of cultivation, demonstrating the convergence of their biogeochemical signatures under standardized farming conditions.

### 3.4. Correlation Analysis

**Figure 5:**
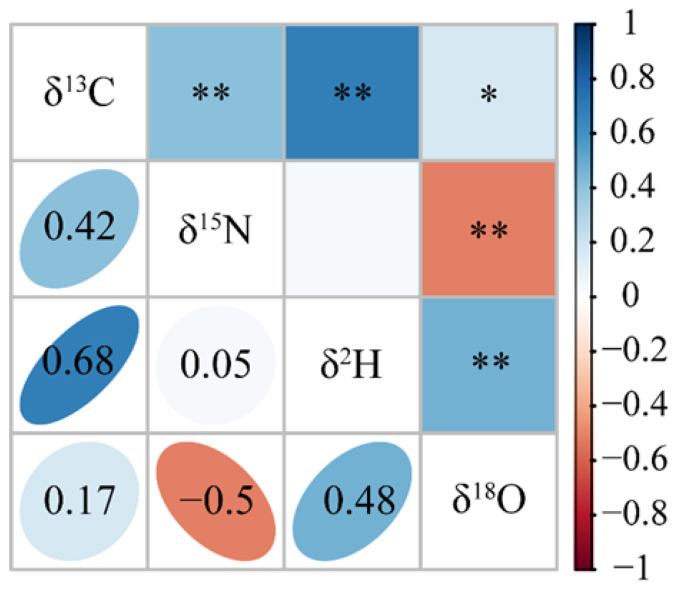
Correlation analysis of stable isotopes in Chinese mitten crab. * Significance level of 0.05; ** significance level of 0.01.

The correlation analysis results (Figure 5) showed that except for the non-significant correlation between *δ*^15^N and *δ*^2^H, all other variables exhibited significant correlations (*p* < 0.05). Specifically, *δ*^13^C and *δ*^18^O demonstrated a significantly positive correlation (*p* < 0.05), while *δ*^13^C with *δ*^15^N, *δ*^13^C with *δ*^2^H, and *δ*^2^H with *δ*^18^O displayed highly significant positive correlations (*p* < 0.01). In contrast, *δ*^15^N and *δ*^2^H showed a highly significant negative correlation (*p* < 0.01).

## 4. Discussion

The stable carbon isotope composition in aquatic products is closely related to feed types [32,33,34], as *δ*^13^C varies with the proportion of C_3_ and C_4_ plants in their diet. *δ*^15^N is influenced not only by the animal-to-plant ratio in feed but also by factors such as soil conditions [35]. The *δ*^2^H and *δ*^18^O values in water bodies vary seasonally and with altitude and latitude, leading to significant differences in these isotopes among aquatic products from distinct environments [36], making them promising for origin tracing. In studies on Italian rainbow trout (*Oncorhynchus mykiss*), *δ*^13^C and *δ*^15^N ratios in fish tissues showed positive correlations with those in feed, while *δ*^2^H and *δ*^18^O correlated with water values. In this study, the gradual increase and subsequent stabilization of *δ*^13^C (*p* > 0.05) may indicate a stable C_3_/C_4_ plant ratio in the feed [37]. The study by Li et al. [38] on Pacific white shrimp (*Litopenaeus vannamei*) further revealed that *δ*^13^C values varied due to salinization-driven enrichment, suggesting that Yangcheng Lake crabs achieve stable *δ*^13^C enrichment through both feed and lake water, making *δ*^13^C a robust geographical tracer for origin authentication. In this study, the diet initially consisted primarily of fish and shellfish, and then shifted during the mid-to-late stages to one dominated by corn and commercial pellet feed. This change led to a gradual increase in *δ*^13^C values during rearing, with stabilization occurring after six months, thus establishing a stable carbon isotopic fingerprint. *δ*^15^N remained at low levels in March when crabs were initially introduced to the lake aquaculture system but exhibited significant fluctuations after one month of farming, likely due to abrupt shifts in nitrogen content between initial feed (e.g., fish, shellfish, or commercial pellet feed) and post-transition diets. Although *δ*^15^N demonstrated lower stability than *δ*^13^C during lake farming, only a transient decline occurred in November. The *δ*^18^O values displayed a distinct pattern of initial decrease followed by an increase, aligning with seasonal dissolved oxygen dynamics in water (lower in summer and higher in winter), which mirrored the *δ*^18^O trends observed in Yangcheng Lake crabs. Given the significant *δ*^18^O variations across different water bodies, this isotope also serves as a reliable origin indicator, though seasonal consistency in sample collection is recommended to minimize confounding effects.

In related studies on other food products, analyses of elemental correlations have also been reported [39,40]. In the study by Tan et al. [40] on rice, significant correlations were observed among nine selected elements, with strong associations detected between nutritional elements and between harmful elements, but no consistent correlations between mineral nutrients and toxic elements. In the mineral element analysis of Yangcheng Lake crabs across an annual cycle [41], multiple groups of nutritional elements (e.g., K-Cu, Mg-K) exhibited significant positive or negative correlations under specific conditions, suggesting synergistic and antagonistic interactions during the absorption and accumulation of environmental elements in the crabs. These inter-element synergies during long-term aquaculture likely contribute to the geographical differentiation. Building on this, the present study explored correlations among stable isotope ratios during the formation of geographical signatures. Results revealed that except for the non-significant correlation between *δ*^15^N and *δ*^2^H, *δ*^13^C and *δ*^18^O showed a significant positive correlation (*p* < 0.05), while all other variable pairs exhibited highly significant correlations (*p* < 0.01). This indicates potential synergistic or antagonistic interactions among *δ*^13^C, *δ*^15^N, *δ*^2^H, and *δ*^18^O during the development of geographical distinctiveness through dietary and environmental influences. Future origin-tracing studies should prioritize precise characterization of stable isotope patterns to enhance the accuracy of geographical authentication.

In studies investigating the annual variations in carapace geometric morphology and multi-mineral elements of Yangcheng Lake farmed crabs [24,41], it was found that carapace morphological stabilization required six months, while mineral element stabilization was achieved within 3–4 months. Similarly, the formation of stable isotopic ‘fingerprint’ characteristics in this study also necessitated a six-month period. Although temporal differences exist in the development of geographical signatures, all stabilization processes were completed prior to the adult crab stage [42], i.e., before market distribution. Therefore, precise characterization of carapace morphology, mineral profiles, and stable isotopic signatures in Yangcheng Lake-originated crabs is critical for effectively protecting the geographical indication status of this iconic product. This method provides a reliable, science-based technique to verify the geographical origin of Chinese mitten crab. It can be effectively used to combat fraudulent practices, such as the mislabeling of cultured crabs as wild-caught or the sale of “bathed crabs” from unknown origins as high-value regional specialties. This protects both consumers’ rights and the economic interests of legitimate producers. The successful delineation of Yangcheng Lake crab’s geographical signatures provides a valuable reference framework for protecting other geographical indication aquatic products, complementing existing origin-tracing theories. This approach could be extended to premium food products and specialty aquatic species, facilitating the construction of integrated databases to advance origin authentication practices across diverse geographical indication systems.

## 5. Conclusions

This study analyzed multiple stable isotope ratios (*δ*^13^C, *δ*^15^N, *δ*^2^H, and *δ*^18^O) in the third pereiopods of Yangcheng Lake Chinese mitten crabs over a one-year aquaculture cycle. Results demonstrated that *δ*^13^C stabilized after prolonged farming, while *δ*^18^O exhibited a distinct seasonal trend of initial decline followed by an increase. Significant correlations were observed among the measured isotope ratios during long-term cultivation. Integrated principal component analysis (PCA) and linear discriminant analysis (LDA) of these isotopic signatures revealed that the geographical isotopic fingerprint of Yangcheng Lake crabs required six months to fully develop, a process dependent on sustained aquaculture conditions. Thus, short-term farmed crabs or counterfeit products falsely marketed as Yangcheng Lake origin cannot replicate these stable isotopic characteristics. The successful identification of third pereiopod isotopic signatures provides an effective tool for safeguarding the geographical indication value of Yangcheng Lake crabs while offering a methodological framework for protecting other globally renowned geographical indication aquatic products through isotopic authentication. This approach further validates the critical role of long-term environmental imprinting in establishing biogeochemical provenance markers.

## Figures and Tables

**Table 1:** Morphometric information of *E. sinensis* populations (mean ± SD).

Sample	Weight (g)	Length (mm)	Width (mm)	Height (mm)	Water Contents (%)
Mar. 2018 (n = 20)	10.81 ± 3.85	25.88 ± 3.10	29.26 ± 3.55	13.42 ± 1.64	53.9 ± 1.4
Apr. 2018 (n = 20)	13.63 ± 3.05	29.36 ± 2.08	32.88 ± 2.44	15.56 ± 1.56	57.4 ± 7.3
May. 2018 (n = 20)	31.14 ± 5.98	38.60 ± 2.18	43.19 ± 2.60	19.95 ± 1.37	66.6 ± 4.7
Jun. 2018 (n = 20)	50.98 ± 13.78	44.73 ± 3.46	50.68 ± 3.93	23.53 ± 1.69	64.1 ± 4.9
Jul. 2018 (n = 20)	70.46 ± 11.84	49.77 ± 2.50	56.37 ± 2.83	27.03 ± 1.59	59.3 ± 1.7
Aug. 2018 (n = 20)	82.67 ± 20.79	52.44 ± 3.73	59.29 ± 4.39	28.64 ± 2.19	61.9 ± 4.3
Sep. 2018 (n = 20)	101.88 ± 13.57	55.58 ± 3.17	63.06 ± 3.72	30.72 ± 1.94	56.9 ± 5.0
Oct. 2018 (n = 20)	116.92 ± 26.73	57.51 ± 3.37	63.96 ± 4.13	31.34 ± 2.09	59.3 ± 3.8
Nov. 2018 (n = 20)	95.21 ± 20.61	54.81 ± 2.63	60.68 ± 3.53	30.06 ± 1.26	58.1 ± 2.4
Dec. 2018 (n = 20)	118.79 ± 23.36	58.91 ± 3.06	65.22 ± 3.63	32.43 ± 1.65	57.0 ± 1.8
Jan. 2019 (n = 20)	130.77 ± 35.67	61.02 ± 4.10	68.02 ± 5.24	34.04 ± 1.85	56.8 ± 3.1
Feb. 2019 (n = 18)	147.52 ± 49.90	63.49 ± 10.35	68.78 ± 7.60	34.79 ± 3.25	59.5 ± 2.0

**Table 2:** Stable isotope ratio (‰) in the third pereiopod.

Sample	*δ*^13^C (‰, VPDB)	*δ*^15^N (‰, Air N_2_)	*δ*^2^H (‰, VSMOW)	*δ*^18^O (‰, VSMOW)
Mar. 2018 (n = 20)	−23.49 ± 0.65	5.91 ± 0.34	−88.03 ± 3.93	20.20 ± 1.13
Apr. 2018 (n = 20)	−22.75 ± 1.34	9.49 ± 1.39	−95.66 ± 8.01	17.18 ± 1.25
May. 2018 (n = 20)	−21.16 ± 0.80	10.86 ± 1.11	−92.56 ± 6.46	16.95 ± 1.07
Jun. 2018 (n = 20)	−20.41 ± 0.77	12.25 ± 0.48	−87.89 ± 4.32	16.40 ± 0.82
Jul. 2018 (n = 20)	−19.40 ± 0.69	11.76 ± 0.30	−85.32 ± 4.81	17.31 ± 0.62
Aug. 2018 (n = 20)	−18.78 ± 2.05	11.72 ± 1.05	−78.80 ± 6.73	17.27 ± 1.08
Sep. 2018 (n = 20)	−17.70 ± 0.65	11.42 ± 0.58	−83.64 ± 5.53	18.16 ± 1.04
Oct. 2018 (n = 20)	−17.02 ± 1.01	10.89 ± 0.54	−75.31 ± 4.70	18.34 ± 1.15
Nov. 2018 (n = 20)	−17.28 ± 0.58	10.19 ± 0.31	−66.08 ± 6.02	19.10 ± 0.69
Dec. 2018 (n = 20)	−17.15 ± 0.58	10.59 ± 0.34	−84.13 ± 5.02	18.67 ± 0.71
Jan. 2019 (n = 20)	−17.22 ± 0.63	11.04 ± 0.23	−68.60 ± 6.12	18.71 ± 0.59
Feb. 2019 (n = 18)	−17.34 ± 0.90	10.52 ± 1.42	−60.53 ± 8.38	19.78 ± 0.65

**Table 3:** Linear discriminant analysis results of linear discrimination based on the third pereiopod stable isotopes.

**Sample**	**Mar. 2018**	**Apr. 2018**	**May. 2018**	**Jun. 2018**	**Jul. 2018**	**Aug. 2018**
discriminant accuracy (%)	100	70	80	85	60	60
**Sample**	**Sep. 2018**	**Oct. 2018**	**Nov. 2018**	**Dec. 2018**	**Jan. 2019**	**Feb. 2019**
discriminant accuracy (%)	70	50	55	80	50	56

## Data Availability

Data will be made available on request.

## References

[1] Herborg L.M., Rushton S.P., Clare A.S., Bentley M.G. (2003). Spread of the Chinese mitten crab (*Eriocheir sinensis*, H. Milne Edwards) in Continental Europe: Analysis of a historical data set. Hydrobiologia.

[2] Rudnick D.A., Hieb K., Grimmer K.F., Resh V.H. (2003). Patterns and processes of biological invasion: The Chinese mitten crab in San Francisco Bay. Basic Appl. Ecol..

[3] Cheng Y., Wu X., Yang X., Hines A.H. (2008). Current trends in hatchery techniques and stock enhancement for Chinese mitten crab, *Eriocheir japonica sinensis*. Rev. Fish. Sci..

[4] Wang J., Xu P., Zhou G., Li X., Lu Q., Liu X., Zhou J., Wang C. (2018). Genetic improvement and breeding practices for Chinese mitten crab, *Eriocheir sinensis*. J. World Aquac. Soc..

[5] Zhang C., Li Q., Wu X., Liu Q., Cheng Y. (2018). Genetic diversity and genetic structure of farmed and wild Chinese mitten crab (*Eriocheir sinensis*) populations from three major basins by mitochondrial DNA COI and Cyt b gene sequences. Mitochondrial DNA Part A.

[6] Wang D., Yao C., Lu Y., Huang D., Li Y., Wu X., Song W., Rao Q. (2025). Origin traceability of Chinese mitten crab (*Eriocheir sinensis*) using multi-stable isotopes and explainable machine learning. Foods.

[7] Mottola A., Piredda R., Catanese G., Lorusso L., Ciccarese P.A., Di G. (2022). Species authentication of canned mackerel: Challenges in molecular identification and potential drivers of mislabelling. Food Control.

[8] Duarte B., Melo J., Mamede R., Carreiras J., Figueiredo A., Fonseca V.F., Sousa M.L., Silva A.B. (2023). In the trail of “Maçã de Alcobaça” protected geographical indication (PGI): Multielement chemometrics as a security and anti-fraud tool to depict clones, cultivars and geographical origins and nutritional value. J. Food Compos. Anal..

[9] Hanner R., Becker S., Ivanova N.V., Steinke D. (2011). FISH-BOL and seafood identification: Geographically dispersed case studies reveal systemic market substitution across Canada. Mitochondrial DNA.

[10] Miller D.D., Mariani S. (2010). Smoke, mirrors, and mislabeled cod: Poor transparency in the European seafood industry. Front. Ecol. Environ..

[11] Lamendin R., Miller K., Ward R.D. (2015). Labelling accuracy in Tasmanian seafood: An investigation using DNA barcoding. Food Control.

[12] Wallstrom M.A., Morris K.A., Carlson L.V., Marko P.B. (2020). Seafood mislabeling in Honolulu, Hawai’i. Forensic Sci. Int. Rep..

[13] Rapa M., Ferrante M., Rodushkin I., Conti M.E. (2024). Safety and quality of grapes: Elemental, isotopic and chemometric analysis from montepulciano d’ Abruzzo PDO chain. Agriculture.

[14] Argolo L.A., Lopez-Fernandez H., Batalha-Filho H., de Mello Affonso P.R.A. (2020). Unraveling the systematics and evolution of the ‘*Geophagus*’ *brasiliensis* (Cichliformes: Cichlidae) species complex. Mol. Phylogenet. Evol..

[15] Regmi B., Douglas M.R., Edds D.R., Douglas M.E. (2021). Geometric morphometric analyses define riverine and lacustrine species flocks of Himalayan snowtrout (Cyprinidae: Schizothorax) in Nepal. Aquat. Biol..

[16] Avigliano E., Domanico A., Sánchez S., Volpedo A.V. (2017). Otolith elemental fingerprint and scale and otolith morphometry in *Prochilodus lineatus* provide identification of natal nurseries. Fish. Res..

[17] Yang Y., Zhang L., Qu X., Zhang W., Shi J., Xu X. (2024). Enhanced food authenticity control using machine learning-assisted elemental analysis. Food Res. Int..

[18] Ji X. (2025). Multielemental analysis using inductively coupled plasma mass spectrometry and optical emission spectroscopy for tracing the geographical origin of food. J. Anal Chem..

[19] Pereira L.A., Santos R.V., Hauser M., Duponchelle F., Carvajal F., Pecheyran C., Bérail S., Pouilly M. (2019). Commercial traceability of *Arapaima* spp. fisheries in the Amazon basin: Can biogeochemical tags be useful?. Biogeosciences.

[20] Zhang X., Cheng J., Han D., Zhao X., Chen X., Liu Y. (2019). Geographical origin traceability and species identification of three scallops (*Patinopecten yessoensis*, *Chlamys farreri*, and *Argopecten irradians*) using stable isotope analysis. Food Chem..

[21] Yin H.M., Huang F., Shen J., Yu H.M. (2020). Using Sr isotopes to trace the geographic origins of Chinese mitten crabs. Acta Geochim..

[22] Sim J., Mcgoverin C., Oey I., Frew R., Kebede B. (2023). Stable isotope and trace element analyses with non-linear machine-learning data analysis improved coffee origin classification and marker selection. J. Sci. Food Agric..

[23] Han C., Li L., Dong X., Gao Q., Dong S. (2022). Current progress in the authentication of fishery and aquatic products using multi-element and stable isotope analyses combined with chemometrics. Rev. Aquac..

[24] Xue J., Liu H., Jiang T., Chen X., Yang J. (2022). Shape variation in the carapace of Chinese mitten crabs (*Eriocheir sinensis* H. Milne Edwards, 1853) in Yangcheng Lake during the year-long culture period. Eur. Zool J..

[25] Xue J., Jiang T., Chen X., Liu H., Yang J. (2022). Multi-mineral element profiles in genuine and “bathing” cultured Chinese mitten crabs (*Eriocheir sinensis*) in Yangcheng Lake, China. Fishes.

[26] Xu Y., Xue J., Liu H., Jiang T., Chen X., Yang J. (2023). Identification of “Bathed” Chinese mitten crabs (*Eriocheir sinensis*) using geometric morphological analysis of the carapace. Fishes.

[27] Zhang W., Xue J., Ma L., Yang J. (2025). Carapace morphological characteristics of Chinese mitten crab (*Eriocheir sinensis*) from emerging origins revealed via geometric morphometrics. Animals.

[28] Xu Y., Xue J., Liu H., Jiang T., Chen X., Yang J. (2024). Elemental and stable isotopic signatures for dynamic traceability of genuine and "bathing" cultured Yangcheng *Eriocheir sinensis* crabs. J. Food Compos. Anal..

[29] Luo R., Jiang T., Chen X., Zheng C., Liu H., Yang J. (2019). Determination of geographic origin of Chinese mitten crab (*Eriocheir sinensis*) using integrated stable isotope and multi-element analyses. Food Chem..

[30] Brand W.A., Coplen T.B., Vogl J., Rosner M., Prohaska T. (2014). Assessment of international reference materials for isotope-ratio analysis (IUPAC Technical Report). Pure Appl. Chem..

[31] Fan Z., Deng Y., Yuan Q., Liu X., Shi H., Feng C., Yang Y., Xu L. (2020). Effect of total dissolved gas supersaturation on the tolerance of grass carp (*Ctenopharyngodon idellus*). Environ. Sci. Eur..

[32] Molkentin J., Lehmann I., Ostermeyer U., Rehbein H. (2015). Traceability of organic fish–Authenticating the production origin of salmonids by chemical and isotopic analyses. Food Control.

[33] Wang Y.V., Wan A.H., Lock E.J., Andersen N., Winter-Schuh C., Larsen T. (2018). Know your fish: A novel compound-specific isotope approach for tracing wild and farmed salmon. Food Chem..

[34] Larsen T., Wang Y.V., Wan A.H. (2022). Tracing the trophic fate of aquafeed macronutrients with carbon isotope ratios of amino acids. Front. Mar. Sci..

[35] Amundson R., Austin A.T., Schuur E.A., Yoo K., Matzek V., Kendall C., Uebersax A., Brenner D., Baisden W.T. (2003). Global patterns of the isotopic composition of soil and plant nitrogen. Global Biogeochem. Cycles.

[36] Mahindawansha A., Jost M., Gassmann M. (2022). Spatial and temporal variations of stable isotopes in precipitation in the mountainous region, North Hesse. Water.

[37] Camin F., Perini M., Bontempo L., Galeotti M., Tibaldi E., Piasentier E. (2018). Stable isotope ratios of H, C, O, N and S for the geographical traceability of Italian rainbow trout (*Oncorhynchus mykiss*). Food Chem..

[38] Li L., Ren W., Dong S., Feng J. (2018). Investigation of geographic origin, salinity and feed on stable isotope profile of Pacific white shrimp (*Litopenaeus vannamei*). Aquac. Res..

[39] Nawaz Z., Kakar K.U., Li X.B., Li S., Zhang B., Shou H.X., Shu Q.Y. (2015). Genome-wide association mapping of quantitative trait loci (QTLs) for contents of eight elements in brown rice (*Oryza sativa* L.). J. Agric. Food Chem..

[40] Tan Y., Sun L., Song Q., Mao D., Zhou J., Jiang Y., Wang J., Fan T., Zhu Q., Huang D. (2020). Genetic architecture of subspecies divergence in trace mineral accumulation and elemental correlations in the rice grain. Theor. Appl. Genet..

[41] Xue J., Jiang T., Chen X., Liu H., Yang J. (2022). Multi-mineral fingerprinting analysis of the Chinese mitten crab (*Eriocheir sinensis*) in Yangcheng Lake during the year-round culture period. Food Chem..

[42] He J., Wu X., Li J., Huang Q., Huang Z., Cheng Y. (2014). Comparison of the culture performance and profitability of wild-caught and captive pond-reared Chinese mitten crab (*Eriocheir sinensis*) juveniles reared in grow-out ponds: Implications for seed selection and genetic selection programs. Aquaculture.

